# Non-coding RNAs as potential mediators of resistance to lung cancer immunotherapy and chemotherapy

**DOI:** 10.32604/or.2024.058256

**Published:** 2025-04-18

**Authors:** JIAHUI WANG, HONGCHENG GE, ZHENGYUAN YU, LINGZHI WU

**Affiliations:** 1 Department of Medical Oncology, The First Affiliated Hospital of Soochow University, Suzhou, 215006, China; 2 The First Clinical College, Zhejiang Chinese Medical University, Hangzhou, 310053, China; 3 Department of Gastroenterology, The First Affiliated Hospital of Zhejiang Chinese Medical University, Hangzhou, 310018, China

**Keywords:** Non-coding RNAs (ncRNAs), Lung cancer, Drug resistance, Immunotherapy, Chemotherapy, Immune checkpoints, Immune escape

## Abstract

Lung cancer is a common cause of cancer-related death globally. The majority of lung cancer patients initially benefit from chemotherapy and immunotherapy. However, as the treatment cycle progresses and the disease evolves, the emergence of acquired resistance leads to treatment failure. Many researches have shown that non-coding RNAs (ncRNAs) not only influence lung cancer progression but also act as potential mediators of immunotherapy and chemotherapy resistance in lung cancer, mediating drug resistance by regulating multiple targets and pathways. In addition, the regulation of immune response by ncRNAs is dualistic, forming a microenvironment for inhibits/promotes immune escape through changes in the expression of immune checkpoints. The aim of this review is to understand the effects of ncRNAs on the occurrence and development of lung cancer, focusing on the role of ncRNAs in regulating drug resistance of lung cancer.

## Introduction

Lung cancer is a common cause of cancer-related deaths globally, with 2 million people diagnosed and 1.76 million dying from the disease each year [1]. The histological classification of lung cancer is mainly divided into non-small cell lung cancer (NSCLC) and small cell lung cancer (SCLC) according to its growth and spreading mode [2]. With the advancement of research, immunotherapy alone or in combination with chemotherapy can significantly improve the survival of patients with advanced lung cancer [3,4]. Immunotherapy targets immune checkpoints with immune-checkpoint inhibitors (ICIs) to activate T cells and exert anti-tumor immunoreactivity [5,6]. Immune checkpoints that mediate tumor immune escape mainly include Programmed cell death protein 1 (PD-1), Programmed death-ligand 1 (PD-L1), and Cytotoxic T lymphocyte-associated antigen 4 (CTLA-4) [7]. The combination of PD-1 and its ligand PD-L1 inhibits the attack of CD8^+^ T cells, which eventually induces tumor cells to evade immune surveillance. Similar to PD-1, CTLA-4 directly obstructs the activation of CD8^+^ T cells by competitively binding to its ligand CD80/CD86, thereby enhancing immune resistance [8–10]. Chemotherapy is the standard treatment of choice for all stages of NSCLC, with cisplatin (DDP) and taxanes as common drugs [11]. For patients with late-stage NSCLC, platinum-containing two-agent chemotherapy (cisplatin or carboplatin in combination with paclitaxel and docetaxel) is a commonly used first-line treatment option. In some clinical studies, the objective remission rate (ORR) has been in the range of 20%–40% [12]. In immunotherapy, for patients with high PD-L1 expression (TPS ≥ 50%), anti-PD-L1 inhibitor monotherapy may result in an ORR of 44.8%. However, for patients with low PD-L1 expression (TPS ≤ 49%), anti-PD-L1 inhibitors may be used in only 15%–25% of cases [13]. Drug resistance is the main cause of adverse reactions to chemotherapy and immunotherapy [14–16], significantly limiting treatment efficacy.

Although ncRNAs don’t encode a protein, they act as regulators in various cancers, regulating cell proliferation, invasion, and metastasis at the transcriptional, translational, and post-translational levels [17–20]. Many studies have shown that the mechanisms by which aberrantly expressed ncRNAs mediate drug resistance are extremely complex. For example, NcRNAs can reduce the sensitivity of the organism to chemotherapeutic drugs by regulating a number of cellular signaling pathways [21]. NcRNAs with miRNA binding sites function as sponging miRNAs, thereby mediating chemotherapy and immune resistance [22]. The involvement of ncRNAs in lung cancer drug resistance may also be through the regulation of mRNA expression of specific genes [23].

PD-1/PD-L1 and CTLA-4 are targets of ncRNAs. NcRNAs promote/block the binding of PD-1 to PD-L1 by up-regulating/down-regulating the expression of PD-1/PD-L1 on cells, resulting in the formation of tumor microenvironment that promotes/suppresses immune escape. An increasing number of ncRNAs have been found to be involved in lung cancer-related pathways, which have potential significance in lung cancer treatment. Therefore, it is necessary to have a more comprehensive understanding of the effects of ncRNAs on lung cancer. This review briefly introduces the regulatory functions of ncRNAs in lung cancer genesis and progression and systematically describes the mechanisms by which ncRNAs regulate drug resistance in lung cancer.

## Impact of ncRNAs on Lung Cancer Progression

### The meaning and classification of ncRNAs

Protein-coding genes make up a small portion of the human genome, and a majority of genes are transcribed into ncRNAs [24]. NcRNAs are divided into different categories according to their size: small ncRNAs mainly include microRNAs (miRNAs), tRNA-derived small RNAs (tsRNAs), and PIWI-interacting RNAs (piRNAs) [25], miRNAs are a class of 21–25 nucleotide-long ncRNAs that mainly interfere with the translation of messenger RNAs (mRNAs) and promote the degradation of mRNAs through base-pairing with the complementary sites of mRNAs of target genes, finally altering the expression of genes [26]. NcRNAs greater than 200 nucleotides in length are long non-coding RNAs (lncRNAs), including subclasses such as circular RNAs (circRNAs) [25]. LncRNAs can affect gene expression by targeting transcription factors, mRNAs, and DNA double-stranded [27,28]. CircRNAs can serve as sponges for miRNAs and RNA-binding proteins (RBPs) and regulate transcription and splicing, playing a key role in gene expression [29,30].

### NcRNAs regulate lung cancer progression

**Figure 1:**
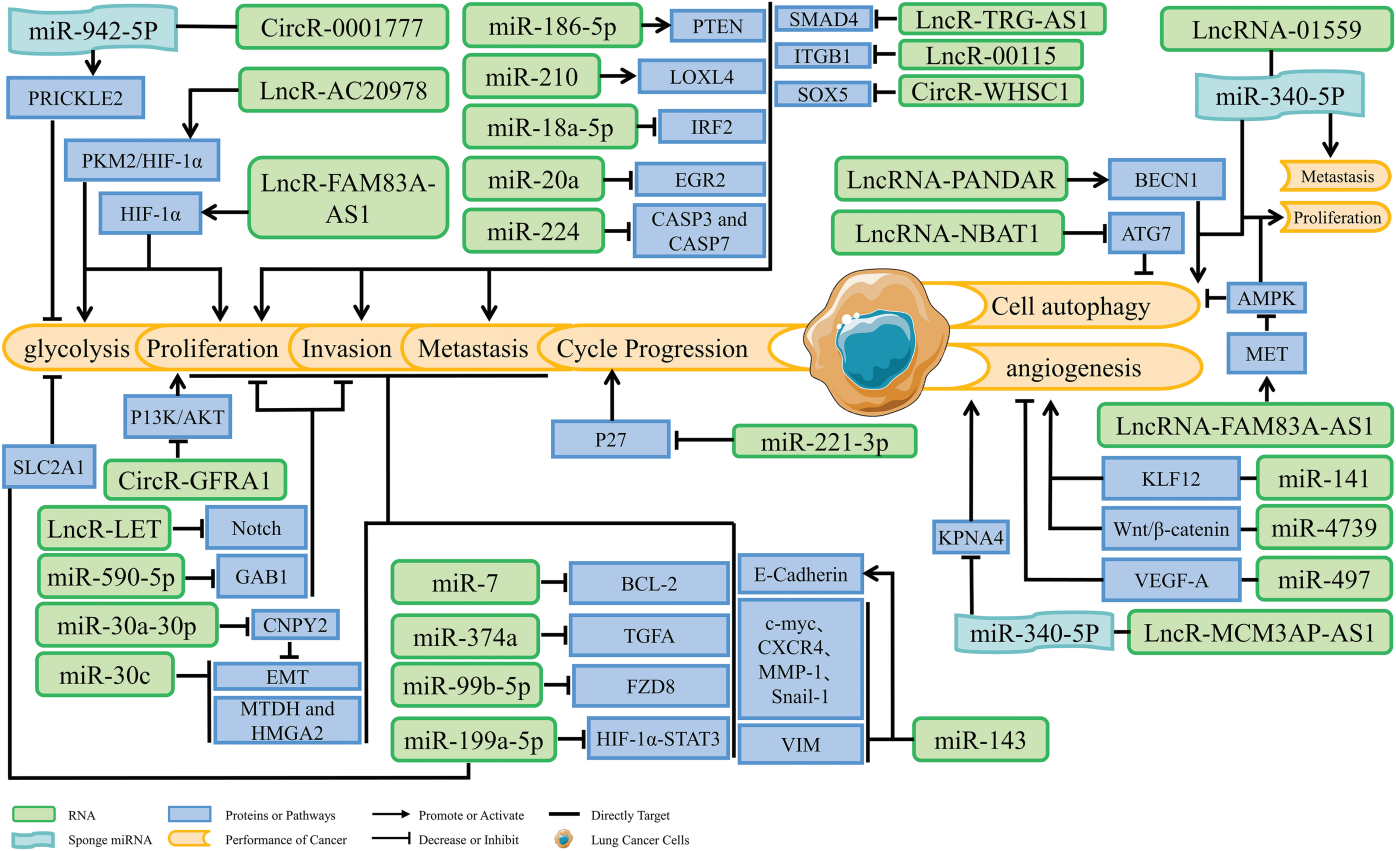
Dual regulation of lung cancer development by non-coding RNAs.

NcRNAs associated with cancer can be broadly classified into two categories: cancer-suppressive ncRNAs and carcinogenic ncRNAs [31,32]. The function of ncRNAs depends on their specific targets. If the target of ncRNAs is an oncogene, ncRNAs can be considered a tumor-inhibiting factor; if the target of ncRNAs is a tumor suppressor gene, ncRNAs can be considered an oncogenic factor [33–35]. Cancer development is usually associated with over expression of oncogene and insufficient expression of tumor suppressor gene. There is increasing evidence that ncRNAs can be involved in lung cancer development and act as an oncogenic factor or tumor-inhibiting factor, positively or negatively regulating the proliferation, invasion, metastasis, angiogenesis, glycolysis, and autophagy of lung cancer cells (Table 1 and Fig. 1).

**Table 1:** NcRNAs positively or negatively regulate lung cancer development

NcRNAs	Location	Mechanisms	Functions	Reference
MiR-224	NSCLC cells	Directly target the caspase-3 and caspase-7 3’UTR, down-regulate the expression of caspase-3 and caspase-7	Promote lung cancer cells proliferation and metastasis	[36]
MiR-20a	NSCLC cells	Directly target EGR2 3’UTR, down-regulate the expression of EGR2	Promote lung cancer cell proliferation, invasion, and metastasis	[37]
MiR-18a-5p	NSCLC cells	Directly target IRF2 3’UTR, down-regulate the expression of IRF2	Promote lung cancer cell proliferation, invasion, and metastasis	[38]
MiR-221-3p	NSCLC cells	Directly target p27 3’UTR, down-regulate the expression of p27	Promote cell cycle progression in lung cancer	[39]
MiR-210	NSCLC cells	Directly target LOXL4	Promote lung cancer cell proliferation, invasion, and metastasis	[40]
MiR-186-5p	NSCLC cells	Directly target PTEN	Promote lung cancer cell proliferation, invasion, and metastasis	[41]
MiR-143	NSCLC cells	Reduce CXCR4, Vimentin, MMP-1, Snail-1, c-Myc expression level, and increasing E-cadherin expression levels	Inhibit lung cancer cell proliferation, invasion, and metastasis	[42]
MiR-30c	NSCLC cells	Inhibit Epithelial-mesenchymal transition (EMT) through down-regulation of MTDH and HMGA2 expression, EMT mediates the development of malignant tumor metastasis	Inhibit lung cancer cell proliferation, invasion, and metastasis	[43,44]
MiR-30a-30p	NSCLC cells	Negatively regulate CNPY2 expression to inhibit EMT	Inhibit lung cancer cell proliferation, invasion, and metastasis	[45]
MiR-199a-5p	NSCLC cells	Down-regulate HIF-1α-STAT3 signaling pathway expression	Inhibit lung cancer cell proliferation, invasion, and metastasis	[46]
MiR-7	NSCLC cells	Directly target BCL-2 3’UTR, down-regulate the expression of BCL-2	Inhibit lung cancer cell proliferation, invasion, and metastasis	[47]
MiR-374a	NSCLC cells	Directly target TFGA 3’UTR, down-regulate the expression of TFGA	Inhibit lung cancer cell proliferation, invasion, and metastasis	[48]
MiR-590-5p	NSCLC cells	Directly target GAB1	Inhibit lung cancer cell proliferation, and invasion	[49]
MiR-141	SCLC cells	Directly target KLF12	Promote lung cancer cell angiogenesis	[50]
MiR-4739	NSCLC cells	Activation of Wnt/β-catenin pathway transduction	Promote lung cancer cell angiogenesis	[51]
MiR-497	NSCLC cells	Directly target Vascular Endothelial Growth factor-A(VEGF-A)	Inhibit lung cancer cell angiogenesis	[52]
MiR-199a-5p	NSCLC cells	Directly target SLC2A1	Inhibit lung cancer cell proliferation and glycolysis	[53]
LncR-LET	NSCLC cells	Inhibition of Notch signaling pathway by down-regulating Notch 1	Inhibition of lung cancer cell proliferation and malignancy	[54,55]
LncR-00115	NSCLC cells	Promote the expression of ITGB1 via sponging miR-607	Promote lung cancer cell proliferation, invasion, and metastasis	[56]
LncR-AC020978	NSCLC cells	Up-regulation of PKM2/HIF-1α axis expression	Promote lung cancer cell proliferation and glycolysis	[57]
LncR-FAM83A-AS1	NSCLC cells	Promotion of HIF-1α transcriptional activity directly	Promote lung cancer cell proliferation and glycolysis	[58]
LncR-MCM3AP-AS1	Lung cancer cells	Sponge miR-340-5p to down-regulate KPNA4 expression	Promote lung cancer cell angiogenesis	[59]
LncR-PANDAR	NSCLC cells	Up-regulation of BECN1 expression	Activation of autophagy in lung cancer cells	[60]
LncR-NBAT1	NSCLC cells	Inhibition of ATG7 transcriptional activity	Inhibit autophagy in lung cancer cells	[61]
LncR-01559	NSCLC cells	Sponge miR-1343-3p	Promote autophagy, proliferation and metastasis of lung cancer cells	[62]
LncR- FAM83A-AS1	NSCLC cells	Up-regulation of MET expression to inhibit AMPK activation	Inhibit autophagy and promote cells proliferation in lung cancer	[63]
CircR-GFRA1	NSCLC cells	Sponge miR-188-3p to activate the PI3K/AKT pathway	Promote lung cancer cells proliferation	[64]
CircR-WHSC1	NSCLC cells	Sponge miR-590-5p to up-regulate SOX5 expression	Promote lung cancer cells proliferation, invasion and metastasis	[65]
CircR-0001777	NSCLC cells	Sponge miR-942-5P to promote PRICKLE2 expression	Inhibit lung cancer cells proliferation, invasion and metastasis and glycolysis	[66]

## The Role of PD-1/PD-L1 in the Tumor Immune Microenvironment

The tumor immune microenvironment is mainly composed of cells such as CD8^+^ T cells, CD4^+^ T cells, dendritic cells (DC), natural killer cells (NK), myeloid-derived suppressor cells (MDSCs), tumor-associated macrophages (TAMs) and regulatory T cells (Tregs) [67,68]. In particular, the number and status of CD8^+^ T cells tend to be positively correlated with the tumor immune response. The current research on immune checkpoints focuses mainly on PD-1 and its ligand PD-L1. PD-1 is a co-inhibitory receptor cell expressed on the surface of T cells after antigen stimulation [69]. PD-L1 is a transmembrane protein that is abundantly expressed in almost all types of cancer cells. The combination of these two proteins can significantly inhibit CD8^+^ T cell activation, creating an immunosuppressive tumor microenvironment [70,71].

## Effect of ncRNAs on the Expression of PD-1/PD-L1

**Figure 2:**
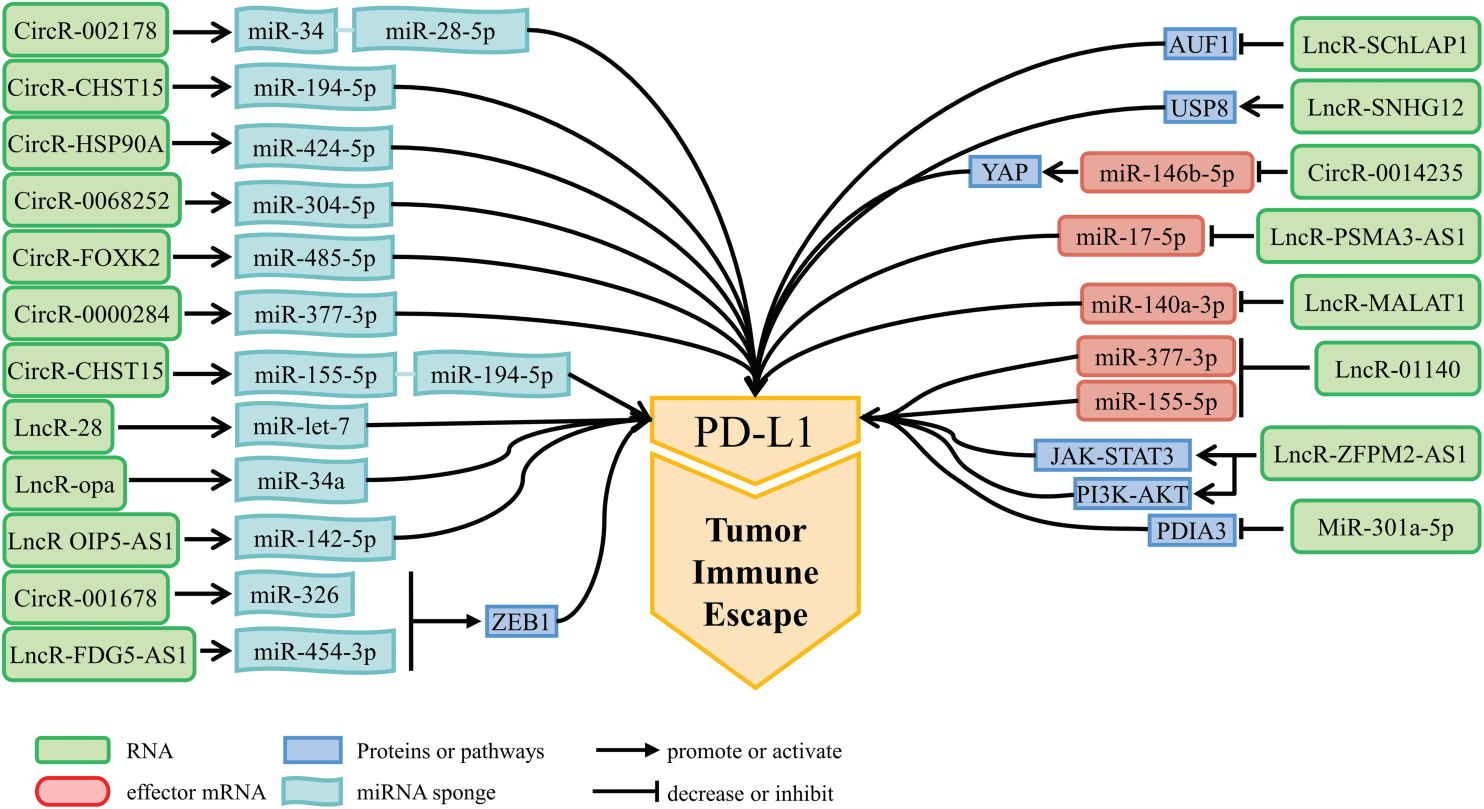
NcRNAs promoting tumor immune escape and their mechanisms of action.

**Figure 3:**
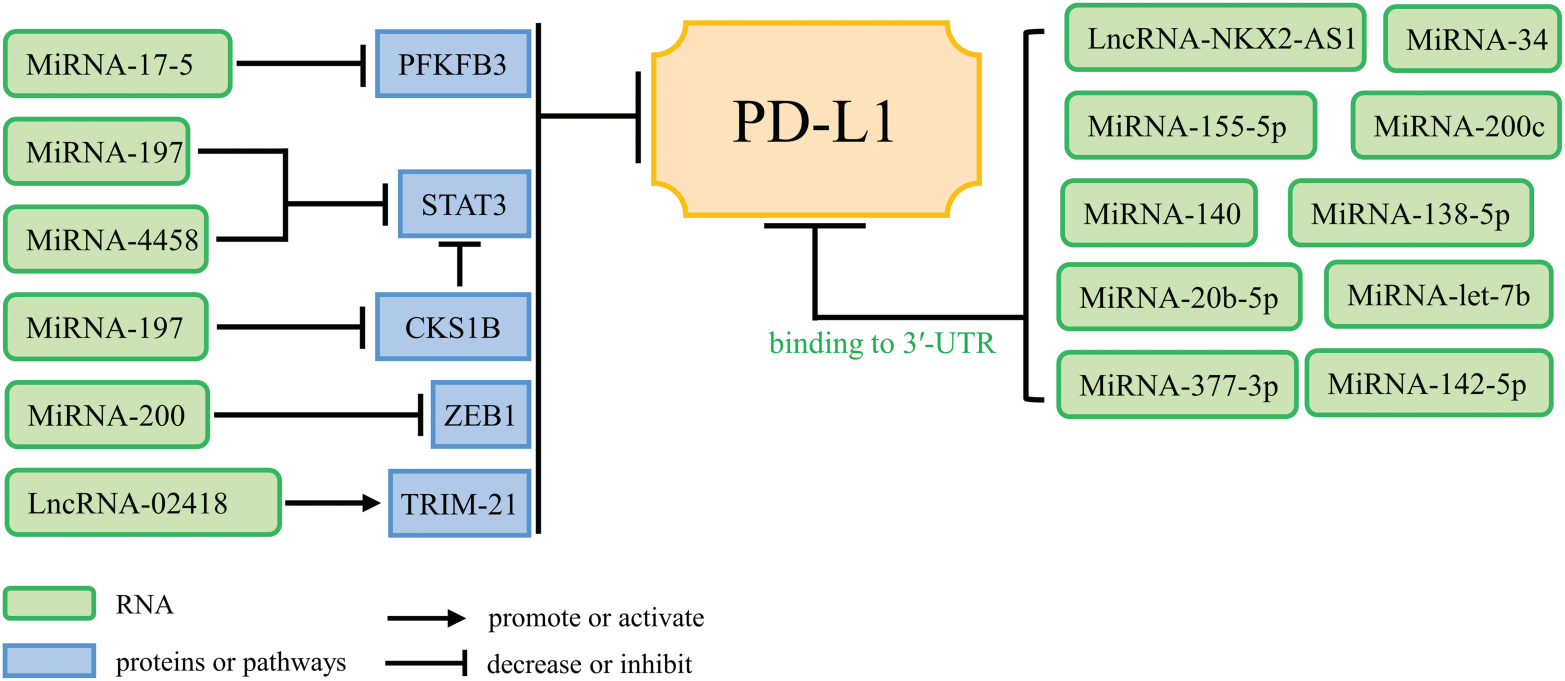
NcRNAs inhibiting tumor immune escape and their mechanisms of action.

Increasing evidence suggests that ncRNAs not only have oncogenic or tumor-suppressive function, but also alter the expression of PD-1/PD-L1 to influence anti-tumor immune response. Tumor-suppressive ncRNAs enhance anti-tumor immune response and promote immune surveillance, but oncogenic ncRNAs can inhibit anti-tumor immunity and promote immune escape [72]. NcRNAs act as immunomodulatory factors (illustrated in Figs. 2 and 3) that promote or inhibit tumor immune escape through modulating downstream signaling pathways or directly acting on PD-1/PD-L1 mRNA expression levels. Tables 2 and 3 briefly summarize the mechanisms of action of ncRNAs to promote or inhibit immune escape in lung cancer.

**Table 2:** NcRNAs promoting tumor immune escape and their mechanisms of action

NcRNAs	Location	Mechanisms	Functions	Reference
CircR-002178	Exosomes of plasma	Sponge miRNA-34a and miRNA-28-5p	Up-regulate PD-1/PD-L1 expression to promote tumor immune escape	[73]
CircR-001678	NSCLC cells	Sponge miR-326 to enhance the expression of ZEB1 transcription, ZEB1 positively correlates with PD-L1 expression	Up-regulate PD-L1 expression to promote tumor immune escape	[74]
CircR-HSP90A	NSCLC cells	Sponge miR-424-5p	Up-regulate PD-L1 expression to promote tumor immune escape	[75]
CircR-0068252	NSCLC cells	Sponge miR-304-5p	Up-regulate PD-L1 expression to promote tumor immune escape	[76]
CircR-FOXK2	NSCLC cells	Sponge miR-485-5p	Up-regulate PD-L1 expression to promote tumor immune escape	[77]
CircR-0014235	NSCLC cells	Down-regulate the target miRNA-146b-5p to promote the expression of YAP, which positively regulates PD-L1 expression	Up-regulate PD-L1 expression to promote tumor immune escape	[78]
CircR-0000284	NSCLC cells	Sponge miR-377-3p	Up-regulate PD-L1 expression to promote tumor immune escape	[79]
CircR-CHST15	Cytoplasm of lung cancer cells	Sponge miR-155-5p and miR-194-5p	Up-regulate PD-L1 expression to promote tumor immune escape	[80]
LncR-FDG5-AS1	NSCLC cells	Sponge miR-454-3p to promote ZEB1 transcription	Up-regulate PD-L1 expression to promote tumor immune escape	[81]
LncR-SChLAP1	NSCLC cells	Block AUF1 binding to the PD-L1 3’UTR	Up-regulate PD-L1 expression to promote tumor immune escape	[82]
LncR-OIP5-AS1	NSCLC cells	Sponge miR-34a	Up-regulate PD-L1 expression to promote tumor immune escape	[83]
LncR-OIP5-AS1	Cancer-associated fibroblasts (CAFs)-derived exosomes	Sponge miR-142-5p	Up-regulate PD-L1 expression to promote tumor immune escape	[84]
LncR-SNHG12	NSCLC cells	Promote USP8 expression by binding to HUR, and USP8-mediated deubiquitination can enhance the stability of PD-L1 expression	Up-regulate PD-L1 expression to promote tumor immune escape	[85]
LncR-PSMA3-AS1	NSCLC cells	Negatively regulate miR-17-5p	Up-regulate PD-L1 expression to promote tumor immune escape	[86]
LncR-MALAT1	NSCLC cells	Negatively regulate miR-140a-3p	Up-regulate PD-L1 expression to promote tumor immune escape	[87]
LncR-01140	Cytoplasm of lung cancer cells	Negatively regulate miR-377-3p and miR-155-5p	Up-regulate PD-L1 expression to promote tumor immune escape	[88]
LncR-ZFPM2-AS1	NSCLC cells	Positively regulate JAK-STAT3 and PI3K-AKT signaling pathways	Up-regulate PD-L1 expression to promote tumor immune escape	[89]
MiR-301a-5p	Membrane and cytoplasm of lung adenocarcinoma cells	Negatively regulate downstream PDIA3	Up-regulate PD-1/PD-L1 expression to promote tumor immune escape	[90]

**Table 3:** NcRNAs inhibiting tumor immune escape and their mechanisms of action

NcRNAs	Location	Mechanisms	Functions	Reference
MiR-17-5	NSCLC cells	Down-regulate 6-phosphofructo-2-kinase (PFKFB3) expression, which is positively correlated with PD-1/PD-L1 expression	Down-regulate PD-1/PD-L1 expression to inhibit tumor immune escape	[91]
MiR-197	NSCLC cells	Inhibit STAT3 phosphorylation, the activation of STAT3 is positively correlated with PD-L1 expression	Down-regulate PD-L1 expression to inhibit tumor immune escape	[92]
MiR-4458	NSCLC cells	Directly target STAT3	Down-regulate PD-L1 expression to inhibit tumor immune escape	[93]
MiR-197	NSCLC cells	Negatively regulate the target CKS1B to down-regulate STAT3 expression	Down-regulate PD-L1 expression to inhibit tumor immune escape	[94]
MiR-155-5p	NSCLC cells	Directly bind to the PD-L1 3’UTR	Down-regulate PD-L1 expression to inhibit tumor immune escape	[95]
MiR-140	NSCLC cells	Directly bind to the PD-L1 3’UTR	Down-regulate PD-L1 expression to inhibit tumor immune escape	[96]
MiR-20b-5p	Lung cancer cells	Directly bind to the PD-L1 3’UTR	Down-regulate PD-L1 expression to inhibit tumor immune escape	[97]
MiR-let-7	Lung cancer cells	Directly bind to the PD-1/PD-L1 3’UTR	Down-regulate PD-1/PD-L1 expression to inhibit tumor immune escape	[98,99]
MiR-138-5p	NSCLC cells	Directly bind to the PD-L1 3’UTR	Down-regulate PD-L1 expression to inhibit tumor immune escape	[100]
MiR-200c	NSCLC cells	Directly bind to the PD-L1 3’UTR	Down-regulate PD-L1 expression to inhibit tumor immune escape	[100]
MiR-142-5p	NSCLC cells	Directly bind to the PD-L1 3’UTR	Down-regulate PD-L1 expression to inhibit tumor immune escape	[84]
MiR-377-3p	Cytoplasm of lung cancer cells	Directly bind to the PD-L1 3’UTR	Down-regulate PD-L1 expression to inhibit tumor immune escape	[88]
MiR-34	NSCLC cells	Directly bind to the PD-L1 3’UTR	Down-regulate PD-L1 expression to inhibit tumor immune escape	[101]
MiR-200	NSCLC cells	Inhibit ZEB1 expression, which is positively correlated with PD-L1 expression	Down-regulate PD-L1 expression to inhibit tumor immune escape	[102]
LncR-02418	NSCLC cells	Facilitate TRIM-21-mediated ubiquitination of PD-L1	Down-regulate PD-L1 expression to inhibit tumor immune escape	[103]
LncR-NKX2-AS1	Lung cancer cells	Negatively regulate the transcriptional activity of the PD-L1 promoter	Down-regulate PD-L1 expression to inhibit tumor immune escape	[104]

## NcRNAs Mediate Resistance to Other Drugs by Regulating PD-1/PD-L1 Expression

PD-1/PD-L1 not only modulates tumor immune escape, but also mediates resistance to chemotherapy and targeted drugs. It has been shown that PD-L1-rich exosomes derived from NSCLC cells reduce the sensitivity of some NSCLC cell subsets to cisplatin by inducing cancer stem cells (CSCs) to maintain their heterogeneity [105,106]. Blocking PD-L1 expression restored the sensitivity of ncRNAs to DDP [106]. Alteration of PD-1/PD-L1 expression is achieved by ncRNAs sponging miRNA and modulating the expression of downstream target genes. DDP is an alkylating agent that cross-links with the DNA of NSCLC cells to form DNA adducts, inducing DNA damage and leading to cell death [107]. CircR-CPA4 sponges miR-let-7 to up-regulate PD-L1 expression in NSCLC, which not only promotes tumor immune escape but also induces DDP resistance [106]. Activated signal transducer and activator of transcription 3 (STAT3) is regulating PD-L1 expression, miR-526b-3p inhibits STAT3 phosphorylation to down-regulate PD-L1 expression, thereby inhibiting immune escape and increasing the sensitivity of the organism to DDP [108]. The opposite of miR-526b-3p is miR-3127-5p, which upregulates PD-L1 expression by promoting STAT3 phosphorylation, induces immune escape and ultimately leads to DDP resistance [109]. LncR-FGD5-AS1 sponges miR-142 to promote PD-L1 expression and subsequently increase DDP resistance [110]. MiR-197 down-regulates STAT3 by negatively regulating the target CDC28 protein kinase regulatory subunit 1B (CKS1B), which inhibits the expression of PD-L1 and increases the sensitivity of the organism to DDP [94]. Furthermore, it has been found that activation of the Phosphatidylinositol 3-kinase-Akt (PI3K-AKT) pathway increased PD-L1 expression and promoted DDP resistance in NSCLC [111], but there is limited research on the upstream mechanisms that regulate the PIK3-AKT/PD-L1 axis. Epithelial growth factor receptor (EGFR) is a transmembrane protein with tyrosine kinase activity, and NSCLC patients with mutations or overexpression of the EGFR kinase structural domain can be targeted by EGFR-tyrosine kinase inhibitor (EGFR-TKI), such as gefitinib [112]. Previous research has shown that PD-L1 expression is significantly increased in gefitinib-resistant tumor cells [113]. Zhang et al. found that PD-L1-promoted resistance to gefitinib in NSCLC cells was induced by activating the transforming growth factor-β (TGF-β)/Smad pathway to induce Epithelial-Mesenchymal Transition (EMT) [114]. In NSCLC, cells that have not undergone EMT are usually more sensitive to gefitinib than those that have experienced EMT [115]. NcRNAs increase gefitinib resistance by activating the PI3K/AKT and Mitogen-activated protein kinase kinase/Extracellular signal-regulated kinase (MEK/ERK) signaling pathways downstream of EGFR [116]. Recent studies have revealed that ncRNAs can also regulate PD-L1 expression to induce gefitinib resistance. CircR-0014235 negatively regulates the target miR-146b-5p to up-regulate Yes-associated protein (YAP) and thereby promotes the expression of PD-L1, which promotes immune escape in NSCLC, and also mediates resistance to gefitinib [78]. Similarly, circR-0091537 induces gefitinib resistance in NSCLC through regulating the miR-520h/YAP/PD-L1 axis [117].

## NcRNAs Mediate Resistance to Anti-PD-1/PD-L1 Inhibitors

Anti-PD-1/PD-L1 inhibitors reduce the expression level of PD-1/PD-L1 or promote the degradation of PD-1/PD-L1 to block the binding of PD-1 with PD-L1, restoring the body’s immune system to recognize and attack tumor cells again [118]. A proportion of patients initially respond to anti-PD-1/PD-L1 therapy, but as time progresses, the tumor recurrence or metastasis, suggesting that the patient has acquired immune resistance [119]. Anti-PD-1/PD-L1 inhibitors resistance is a major cause of immunotherapy failure. Therefore, the research on the molecular mechanism of anti-PD-1/PD-L1 inhibitor resistance is important for improving the prognosis of patients. The mechanisms by which ncRNAs modulate anti-PD-1/PD-L1 inhibitors sensitivity involve miRNA sponge effect, changes in the expression levels of specific genes and signaling pathways that are closely associated with lung cancer development (such as the Transforming growth factor-β/SMAD family (TGF-β/SMAD) and Phosphatase and tensin homolog (PTEN) signaling pathways). CircR-CELF1 plays an important role in lung cancer development and anti-PD-1 inhibitor resistance. CircR-CELF1 enhances NSCLC cell proliferation, metastasis and invasion by sponging miR-491-5P and promoting EGFR expression. Moreover, the expression of circR-CELF1/EGFR is negatively correlated with CD8^+^ T cells in NSCLC, and the up-regulation of circR-CELF1 facilitates the tumor immune escape and resistance to anti-PD-1 inhibitor through reducing the number of CD8^+^ T cells [120].

CircR-ASCC3 plays an oncogene role in NSCLC and is upregulated in anti-PD-1 inhibitor-resistant NSCLC cells. CircR-ASCC3 sponges miR-432-5p to promote Complement component 5a (C5a) expression, high C5a expression promotes EMT transformation and M2 type macrophage (M2-type) tumor-associated macrophages expression through depletion of CD8^+^ T cells, and ultimately enhances NSCLC progression and immune escape [121]. It has been shown that cytokine secretion by M2-type tumor-associated macrophages induces immune resistance to drugs [122]. Therefore circR-ASCC3 promotes anti-PD-1 inhibitor resistance through shaping the tumor immunosuppressive microenvironment. Coactivator-associated arginine methyltransferase 1 (CARM1), as a negative regulator of anti-tumor immunity, was found to down-regulate the expression of CD8^+^ T cells, dendritic cells (DCs) and natural killer cells (NK cells) and inhibit γ-interferon (IFN-γ) signaling, leading to tumor immune escape and immune drug resistance [123]. In NSCLC, circR-HMGB2 up-regulates the expression of the downstream molecule CARM1 by sponging miR-181a-5p [124]. Thus, the overexpression of circR-HMGB2 induces an immunosuppressive microenvironment in NSCLC and mediates anti-PD-1 inhibitor resistance [124].

CircR-0000190 is significantly overexpressed in anti-PD-L1 inhibitor-resistant NSCLC cells. PTEN is a target of miR-142-5p, circR-0000190 improves PTEN expression and inhibits the PI3K/AKT signaling pathway through down-regulation of miR-142-5p, which ultimately enhances the immune escape and immune resistance of PD-L1-mediated NSCLC cells [125,126].

MiR-326 is considered as a tumor suppressor, which is significantly down-regulated in anti-PD-1 inhibitor-resistant lung cancer cells. Polio virus receptor-related protein 1/Necl-5 (CD155) is an adhesion molecule that contributes to the proliferation, invasion and metastasis of tumor cells through multiple pathways, and also mediates the body’s immune response [127]. CD155 binds to T cell immunoreceptors (TIGIT) on NK cells and CD8^+^ T cells, which can inhibit the activity of NK cells and CD8^+^ T cells, resulting in tumor immune escape [127–129]. The IFN-γ-activated miRNA-326 binds directly to the CD155 3’UTR to negatively regulate CD155 expression, indicating that miR-326 restores the activity of NK cells and CD8^+^ T cells, and increases the sensitivity of lung cancer cells to anti-PD-1 inhibitor [130].

CircR-DENND2D is up-regulated in anti-PD-1/PD-L1 inhibitor-sensitive NSCLC cells. Serine/threonine kinase 11 (STK11) acts as a tumor-inhibiting factor in NSCLC to inhibit lung cancer progression through regulation of cell metabolism and proliferation [131]. Lack of STK11 expression enhances neutrophil recruitment that inhibits CD8^+^ T cells and increases the expression of tumor cytokines, which reduces the efficacy of CD8^+^ T cells in anti-tumor immunity, and eventually promotes resistance to anti-PD-1/PD-L1 inhibitor [132,133]. CircR-DENND2D down-regulates miR-130b-3p to promote STK11 expression, leading to enhancement of CD8^+^ T cells activity, inhibition of tumor immune escape, and reduction of anti-PD-1/PD-L1 inhibitor resistance in NSCLC [134].

CircR-FGFR1 is overexpressed in anti-PD-1 inhibitor-resistant lung cancer cells. Circular RNA related to fibroblast growth factor receptor 1 and C-X-C chemokine receptor type 4 (CircR-FGFR1/CXCR4) expression is negatively correlated with CD8^+^ T cells [135]. CircR-FGFR1 sponges miR-381-3p to up-regulate the expression of CXCR4, thereby decreasing the number and activity of CD8^+^ T cells, and promoting tumor immune suppression and resistance to anti-PD-1 inhibitors [135].

CircR-0003222 is regarded as an oncogenic factor. MiR-527 adversely modulates the TGF-β/SMAD signaling pathway and inhibits NSCLC proliferation and invasion with EMT [136]. CircR-0003222 directly up-regulates PD-L1 expression and sponges miR-527 to activate the Transforming growth factor-β/SMAD family (TGF-β/SMAD) signaling pathway, ultimately promoting lung cancer progression and anti-PD-L1 inhibitor resistance [137].

LncR-XIST is regarded as an oncogenic factor with a promotive role in lung cancer development. Long non-coding RNA X-inactive specific transcript (LncRNA-XIST) down-regulates the target miR-34a-5p to increase the expression of PD-L1 and inhibit the immune function of CD8^+^ T cells, which promotes the growth, migration and invasion of lung cancer, possibly mediating the resistance to anti-PD-L1 inhibitors [138].

LncR-02418 acts as a negative regulator of PD-L1 expression and promotes Tripartite motif-containing protein 21 (TRIM-21)-mediated ubiquitination of PD-L1, which results in up-regulation of CD8^+^ T cells expression and increased sensitivity of NSCLC cells against PD-L1 inhibitors [103].

Lung cancer stem cells have been shown to possess the biological properties of stem cells, such as self-renewal and differentiation, and have potential significance for tumor immune escape and drug resistance [139]. High expression of lncR-AC026356.1 positively correlates with T cells depletion that maintains NSCLC cancer stem cell (CSCs) properties through activation of the Wnt/β-catenin signaling pathway, with the result of inhibiting NSCLC cell sensitivity to anti-PD-1/PD-L1 inhibitors [140,141]. With similar effect to lncR-AC026356.1 is lncR-PKMYT1AR, which sponges miR-485-5p to up-regulate the expression of protein kinase PKMYT1. PKMYT1 inhibits Beta-transducin repeat-containing protein 1 (β-TrCP1)-mediated ubiquitination of catenin proteins, leading to the maintenance of the properties of CSCs in NSCLC, and the promotion of immune escape and drug resistance [142]. Interestingly, it was shown that miR-34a acts very distinctly from lncR-AC026356.1. MiR-34a acts as a tumor suppressor, directly targeting Cluster of differentiation 44 (CD44) to inhibit the growth of NSCLC cells and CSCs, and may suppress the occurrence of immune resistance [143].

MiR-125b-3p expression is markedly up-regulated in anti-PD-1 inhibitor-resistant NSCLC cells. The high expression of miR-125b-3p as a negative regulator of T cells significantly inhibits T cell activation, which may confer immunological resistance to NSCLC cells [144].

Studies have shown that aberrant expression of ncRNAs is associated with drug resistance. NcRNAs are involved in the decrease of cytotoxic T cells (CTLs), the lack of sensitivity of IFN-γ signaling, overexpression or loss of PD-1 on the surface of T cells and PD-L1 on the surface of tumor cells, resulting in the development of immune drugs resistance in the organism. There are significant differences in the expression levels of ncRNAs in immune-resistant cells and immune-sensitive cells in lung cancer. Therefore, ncRNAs can be used as a target to regulate immune sensitivity.

## Effect of ncRNAs on the Expression of CTLA-4 Immune Checkpoint

CTLA-4 is a negative regulator of T cells activation [145], which improves regulatory T cells (Tregs)-mediated immunosuppression by suppressing CTLs immune function, eventually inducing tumor immune escape and drug resistance [146–148]. NcRNAs directly or indirectly regulate the expression of CTLA-4, which significantly affects the immunotherapy efficacy. Anti-CTLA-4 immunotherapy mainly enhances anti-tumor immune response through the elimination of Tregs [149,150]. In colorectal cancer, up-regulation of phosphoglycerate translocase-1 (PGAM1) by circR-QSOX1 leads to over-catalysis of aerobic glycolysis and lactate accumulation, resulting in the promotion of Treg cell-mediated immune suppression and anti-CTLA-4 immune resistance [151]. In prostate cancer, miR-9-3p positively regulates CTLA-4 expression [152]. LncR-MIR22HG inhibits CTLA-4 through down-regulation of miR-9-3p, thereby improving the efficacy of immunotherapy [152]. It has been found that NSCLC patients with high CTLA-4 expression have a poor prognosis, indicating that CTLA-4 may be a potential target for tumor immunotherapy [153]. MiR-33a and CTLA-4 expression are negatively correlated in lung cancer, high expression of miR-33a can inhibit CTLA-4 to improve lung cancer survival apparently [154].

## NcRNAs and DDP Resistance

**Figure 4:**
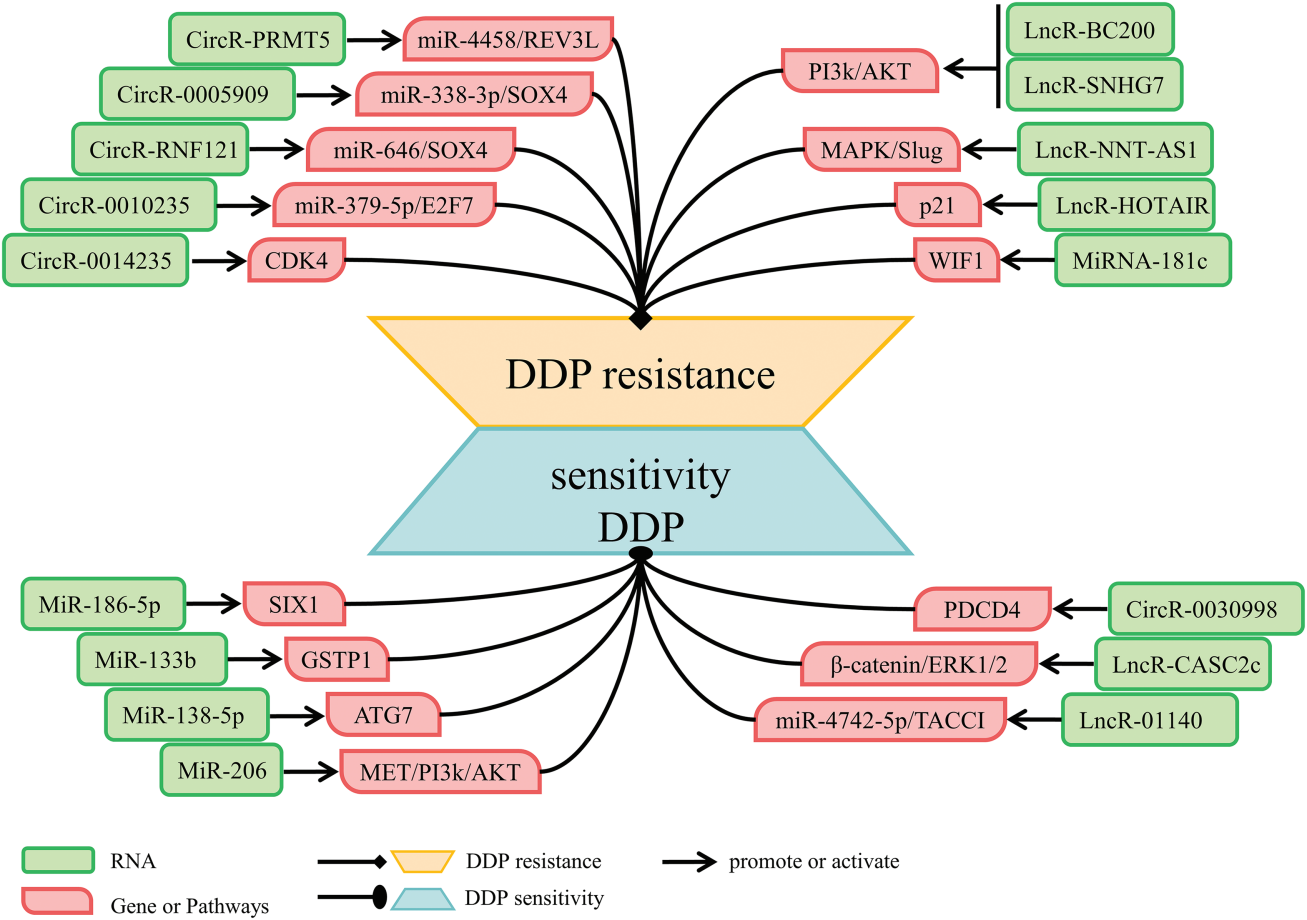
NcRNAs that regulate DDP resistance.

DDP, as the most fundamental chemotherapeutic drug in the treatment of lung cancer [155], can inhibit DNA replication, destroy the structure of cell membrane and promote apoptosis of tumor cells [156], which is widely used in the treatment of lung cancer [157]. However, DDP resistance leads to tumor progression and recurrence and is a major cause of chemotherapy failure [16]. Mechanisms leading to DDP resistance are influenced by multiple factors [158]. Research has found that the expression levels of ncRNAs varies according to their DDP treatment efficacy. Hu et al. found that the expression of 1543 lncRNAs and 1713 mRNAs differed in DDP-sensitive NSCLC cells compared with DDP-resistant NSCLC cells [159]. NcRNAs regulate the expression of genes with specific functions (for example: DNA damage repair, cancer cell stemness, apoptosis, autophagy, EMT), and cancer development-associated Mitogen-activated protein kinase/Snail family transcriptional repressor 2 (MAPK/Slug), PI3K/AKT, and Wnt/β-catenin signaling pathways to alter the therapeutic effect of DDP. Mechanisms that involved ncRNAs and DDP resistance are shown in Table 4 and Fig. 4.

**Table 4:** NcRNAs that regulate DDP resistance

NcRNAs	Location	Genes and pathways	Functions	Reference
LncR-HOTAIR	NSCLC cells	p21	DDP resistance	[160]
LncR-NNT-AS1	NSCLC cells	MAPK/slug pathway	DDP resistance	[161]
LncR-BC200	NSCLC cells	PI3k/AKT pathway	DDP resistance	[162]
LncR-SNHG7	NSCLC cells	PI3K/AKT pathway	DDP resistance	[163]
LncR-01140	NSCLC cells	miR-4742-5p/TACCI pathway	DDP sensitivity	[164]
LncR-CASC2c	NSCLC cells	ERK1/2 and β-catenin pathway	DDP sensitivity	[165]
CircR-0014235	NSCLC cells	miR-520-5p/CDK4 pathway	DDP resistance	[166]
CircR-0005909	NSCLC cells	miR-338-3p/SOX4 pathway	DDP resistance	[167]
CircR-RNF121	NSCLC cells	miR-646/SOX4 pathway	DDP resistance	[168]
CircR-0010235	NSCLC cells	miR-379-5p/E2F7 pathway	DDP resistance	[169]
CircR-PRMT5	NSCLC cells	miR-4458/REV3L pathway	DDP resistance	[170]
CircR-0030998	NSCLC cells	PDCD4	DDP sensitivity	[171]
MiR-181c	NSCLC cells	WIF1	DDP resistance	[172]
MiR-186-5p	NSCLC cells	SIX1	DDP sensitivity	[173]
MiR-133b	NSCLC cells	GSTP1	DDP sensitivity	[174]
MiR-138-5p	NSCLC cells	ATG7	DDP sensitivity	[175]
MiR-206	NSCLC cells	MET, PI3k/AKT pathway	DDP sensitivity	[176,177]

### Up-regulated ncRNAs in DDP resistance

LncR-HOTAIR is significantly upregulated in DDP-resistant NSCLC cells. P21, a cell cycle protein-dependent kinase inhibitor, is overexpressed in response to DNA damage, leading to cell cycle arrest and inhibition of proliferation [178]. Repair of DNA damage in tumor cells is a cellular phenotype mediating DDP resistance [179]. LncR-HOTAIR down-regulates p21 expression to repair DNA damage, inducing DDP resistance [160]. LncR-NNT-AS1 is overexpressed in DDP-resistant NSCLC cells. LncR-NNT-AS1 activates the MAPK/Slug pathway to suppress apoptosis and enhance cell cycle progression and proliferation, resulting in DDP resistance [161]. LncR-BC200, as an oncogenic factor, is up-regulated in DDP-resistant NSCLC cells. Aberrant activation of PI3K/AKT pathway promotes NSCLC cell proliferation, invasion and metastasis, and is closely related to DDP resistance [180]. LncR-BC200 positively regulates the PI3K/AKT pathway, suggesting that lncR-BC200 mediates DDP resistance [162]. CircR-0014235 is notably upregulated in NSCLC progression and DDP resistance. Cyclin-dependent kinase 4 (CDK4) promotes NSCLC cell cycle progression and suppresses apoptosis [181]. CircR-0014235 enhances DDP resistance and malignant behavior in NSCLC cells via upregulation of CDK4 expression [166]. Tumor cells with stemness are resistant to DDP treatment and are associated with DDP resistance. CircR-0005909 is overexpressed in DDP-resistant NSCLC cells. CircR-0005909 induces DDP resistance through sponging miR-338-3p to upregulate the expression of SOX4 [167]. Studies have shown that in various cancers SOX4 activates the TGF-β signaling pathway to induce the EMT of cancer cells, thus maintaining the stemness characteristics of cancer cells [182–185]. CircR-RNF121 enhances DDP resistance in NSCLC cells via the miR-646/SOX4 axis [168]. LncR-SNHG7 is highly expressed in NSCLC cells. Knockdown of lncR-SNHG7 promoted apoptosis and inhibited cell proliferation through down-regulation of the PI3K/AKT pathway, indicating that the PI3K/AKT pathway mediated lncR-SNHG7-induced DDP resistance [163]. CircR-0010235 is overexpressed in DDP-resistant lung cancer cells. E2F transcription factor 7 (E2F7) is considered to be an oncogene that inhibits apoptosis and promotes proliferation and metastasis of lung cancer cells [186,187]. CircR-0010235 sponges downstream miR-379-5p to improve E2F7 expression, resulting in tumor growth and DDP resistance [169]. CircR-PRMT5 is obviously up-regulated in DDP-resistant NSCLC cells, which accelerates tumor growth and reduces the sensitivity of NSCLC cells to DDP. It has been shown that knockdown of DNA polymerase zeta catalytic subunit (REV3L) expression can enhance DDP-mediated DNA damage in NSCLC cells, thereby increasing DDP sensitivity [188]. CircR-PRMT5 sponges miR-4458 to upregulate REV3L, inducing DDP resistance in NSCLC cells [170]. The abnormal activation of Wnt/β-catenin signaling pathway facilitates tumor growth and inhibits the killing of tumor cells by chemotherapy [189,190]. MiR-181c acts as an oncogenic factor and activates the Wnt/β-catenin pathway by targeting Wnt inhibitory factor 1 (WIF1), which subsequently promotes DDP resistance in NSCLC cells [172].

### Down-regulated ncRNAs in DDP resistance

LncR-01140 is downregulated in DDP-resistant NSCLC cells. MiR-4742-5p acts as an oncogenic factor that stimulates cell invasion and suppresses cell apoptosis [164]. LncR-01140 upregulates TACCI expression through sponging miR-4742-5p to restrain NSCLC progression and DDP resistance [164]. LncR-CASC2c exerts a tumor suppressor role that is significant in inhibiting tumor progression and DDP-resistant NSCLC cells. LncR-CASC2c inhibits NSCLC cells proliferation and metastasis by down-regulating ERK1/2 and β-catenin pathways and increases DDP sensitivity of NSCLC cells [165]. CircR-0030998 is low expressed in DDP-resistant NSCLC cells. Programmed cell death 4 (PDCD4) serves as a tumor suppressor in NSCLC that enhances the sensitivity of NSCLC cells to DDP [191,192]. CircR-0030998 increases PDCD4 expression to inhibit DDP drug resistance [171]. The research found that Aurora kinase B (AURBK) plays a role in enhancing the repair of DNA damage, miR-486-5p down-regulated AURBK to suppress DNA damage repair and attenuate the resistance of NSCLC cells to DDP [193]. MiR-186-5p is low expressed in NSCLC. The elevated miR-186-5p expression inhibits NSCLC cell proliferation, invasion, metastasis and resistance to DDP via targeting Sine oculis homeobox homolog 1 (SIX1) [173]. Similarly, miR-133b can directly target Glutathione S-transferase pi 1 (GSTP1) to reverse DDP resistance [174]. Autophagy is an important mechanism involved in chemotherapy resistance [194,195]. Tripartite motif-containing protein 65 (TRIM65) plays an important role in cellular autophagy [175]. The expression of miR-138-5p is markedly reduced in DDP-resistant NSCLC cells. Knockdown of TRIM65 can elevate the expression of miR-138-5p to target Autophagy-related protein 7 (ATG7), resulting in the inhibition of cellular autophagy and enhancement of DDP sensitivity in NSCLC cells [175]. MiR-206 acts as a tumor-inhibiting factor and is obviously down-regulated in DDP-resistant NSCLC cells. MiR-206 targets met proto-oncogene receptor tyrosine kinase (MET) and suppresses its activation of the downstream PI3K/AKT signaling pathway to reduce the incidence of EMT and DDP resistance [176,177].

## NcRNAs and Taxanes Drugs Resistance

**Figure 5:**
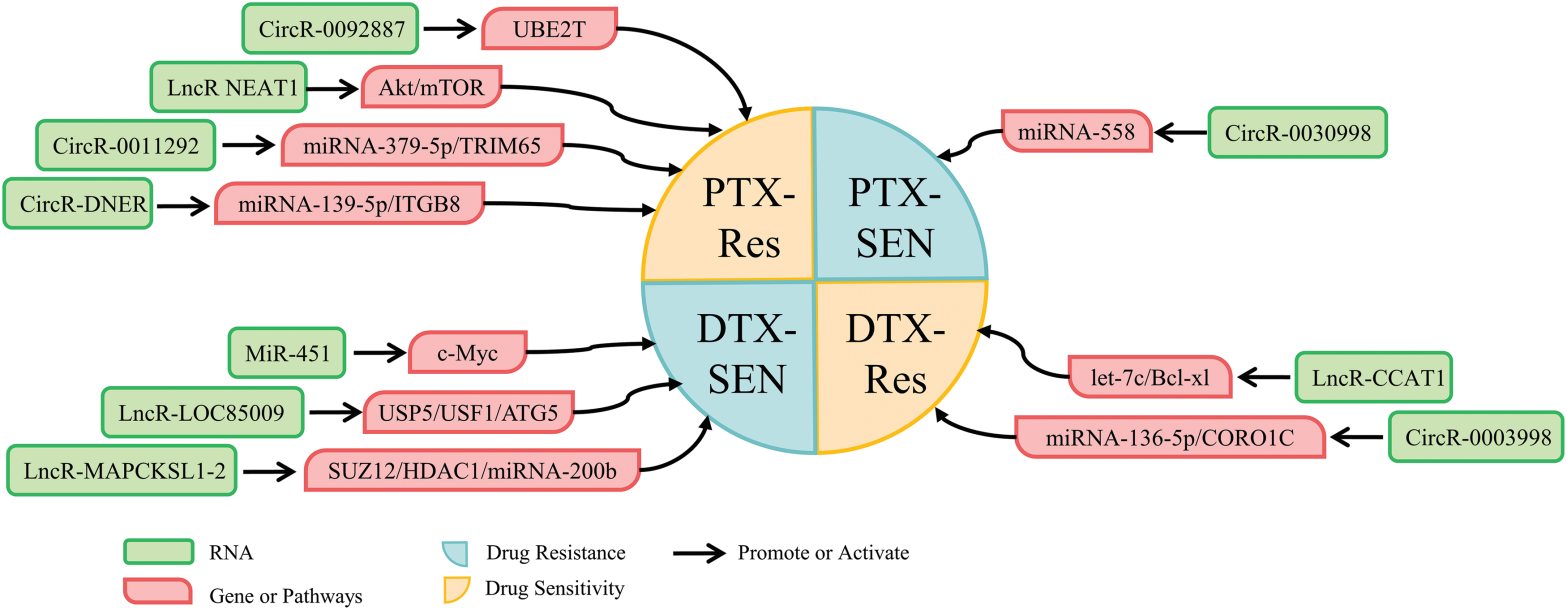
NcRNAs that regulate PTX and DTX drugs resistance.

Taxanes drugs a first-line treatment option for patients with advanced NSCLC [196,197]. The most common taxanes drugs are paclitaxel (PTX) and docetaxel (DTX), but resistance to taxanes drugs is a major obstacle in the treatment of lung cancer [21,198]. Tian et al. found that PTX-resistant NSCLC cells showed a >3-fold difference in the expression of 1154 lncRNAs and 1733 mRNAs when compared to PTX-sensitive NSCLC cells [199]. In lung cancer, ncRNAs have been shown to be a key target for inducing resistance to PTX and DTX. NcRNAs mediating taxanes sensitivity focus on the regulation of cancer cells proliferation and apoptosis. The ncRNAs associated with modulating the sensitivity of lung cancer to taxanes drugs are summarized in Table 5 and Fig. 5.

**Table 5:** NcRNAs that regulate PTX and DTX drugs resistance

NcRNAs	Location	Genes and pathways	Function	Reference
CircR-0011292	NSCLC cells	miR-379-5p/TRIM65 pathway	PTX resistance	[200]
CircR-DNER	Lung cancer cells	miR-139-5p/ITGB8 pathway	PTX resistance	[201]
CircR-0092887	NSCLC cells	UBE2T	PTX resistance	[202]
CircR-0030998	NSCLC cells	miR-558	PTX sensitivity	[203]
CircR-0003998	NSCLC cells	miR-136-5p/CORO1C pathway	DTX resistance	[204]
LncR-NEAT1	NSCLC cells	Akt/mTOR pathway	PTX resistance	[205]
LncR-LOC85009	NSCLC cells	USP5/USF1/ATG5	DTX sensitivity	[206]
LncR-CCAT1	NSCLC cells	let-7c/Bcl-xl pathway	DTX resistance	[207,208]
LncR-MAPCKSL1-2	NSCLC cells	SUZ12/HDAC1/miR-200b pathway	DTX sensitivity	[209]
MiR-451	NSCLC cells	c-Myc	DTX sensitivity	[210]

### NcRNAs and PTX drug resistance

PTX is an anti-microtubule drug, its cytotoxicity by binding to microtubules and inhibiting microtubule depolymerization, thus inhibiting mitosis and promoting apoptosis in tumor cells [211,212]. CircR-0011292 can function as a sponge for miR-379-5p. TRIM65 acts as an oncogenic factor, promotes NSCLC cells proliferation, metastasis and cycle progression, as well as inhibits cells apoptosis [213]. CircR-0011292 leads to PTX resistance in NSCLC by reducing the inhibition of TRIM65 expression caused with miR-379-5p [200]. Likewise, circR-DNER sponges miR-139-5p to upregulate Integrin subunit beta 8 (ITGB8) expression, which contributes to lung cancer progression and PTX resistance [201]. CircR-0092887 is notably upregulated in PTX-resistant NSCLC cells. Knockdown of circR-0092887 down-regulates UBE2T expression, resulting in PTX-resistant NSCLC cells regaining the PTX sensitivity [202]. LncR-NEAT1 is high expressed in PTX-resistant NSCLC cells and mediates PTX resistance through activation of the Protein kinase B/mammalian target of rapamycin (Akt/mTOR) pathway [205]. In addition, circR-0030998 serves as a tumor suppressor and is low expressed in PTX-resistant NSCLC cells. CircR-0030998 acts as a sponge for miR-558 and down-regulates miRNA-558 expression to inhibit tumor deterioration and PTX resistance [203].

### NcRNAs and DTX drug resistance

DTX is an analogue of PTX, which shows greater affinity for the binding site of microtubule proteins compared to PTX [214]. DTX binds to the β-subunit of microtubule proteins to interrupt the mitotic cycle and cause cells death [215,216]. More and more studies show that autophagy is a potential target for reversing chemotherapy resistance [217–219]. Autophagy-related protein 5 (ATG5) is associated with autophagy. Knockdown of ATG5 inhibits autophagy, leading to up-regulation of Vpr-binding protein (VPRPB) expression [220]. LncR-LOC85009 is low expressed in DTX-resistant NSCLC cells. LncR-LOC85009 competitively binds to ubiquitin-specific peptidase 5 (USP5) in order to destabilize the upstream transcription factor 1 (USF1) protein, which inactivates ATG5 transcription, inhibits autophagy and reverses DTX resistance in NSCLC cells [206]. MiR-451 is markedly down-regulated in DTX-resistant NSCLC cells. MiR-451 targets c-Myc to induce the transformation of NSCLC cells with EMT phenotype into mesenchymal-epithelial transition (MET) phenotype, resulting in the restoration of DTX sensitivity in NSCLC cells [210]. EMT-mediated metastasis has been shown to be involved in chemotherapy resistance [221–223]. LncR-CCAT1 is regarded as an oncogenic factor and is noticeably up-regulated in DTX-resistant NSCLC cells. Long non-coding RNA colon cancer-associated transcript 1 (LncR-CCAT1) induces NSCLC cells to acquire an EMT phenotype in association with DTX-resistance by sponging let-7c to promote B-cell lymphoma-extra large (Bcl-xl) [207,208]. LncR-MAPCKSL1-2, considered a tumor-inhibiting factor, is decreased in expression in DTX-resistant NSCLC cells. The knockdown of miR-200b enhances cells proliferation and induces DTX resistance [224]. LncR-MAPCKSL1-2 promotes Suppressor of zeste 12 homolog (SUZ12) binding to Histone deacetylase 1 (HDAC1) to repress HDAC1 transcription, eventually upregulates miR-200b expression and attenuates DTX resistance in NSCLC cells [209]. CircR-0003998 is overexpressed in DTX-resistant NSCLC cells. CircR-0003998 acts as a sponge for miR-136-5p, leading to the overexpression of Coronin 1C (CORO1C), which inhibits apoptosis and promotes DTX resistance [204].

## Conclusions

With the continuous advancement of research, ncRNAs have become a hot topic in recent years. Previous studies have shown that ncRNAs are involved in various pathophysiological changes in lung cancer as oncogenic or tumor-inhibiting factors [225–227], but also as diagnostic biomarkers for lung cancer [228], and have been shown to have meaning in predicting the efficacy of immunotherapy [229]. As mentioned earlier, ncRNAs mediate resistance to immunotherapy and chemotherapy by modulating multiple targets and pathways. Therefore, targeted ncRNA therapies are also gradually demonstrating therapeutic potential. The therapies can be used to inhibit lung cancer progression and improve treatment efficacy through miRNA sponges, ASO, CRISPR/Cas9 gene editing, exosomes and more [230–232]. Although this review focuses on the induction of drug resistance by ncRNAs through sponge miRNAs, experiments have been conducted to show that ncRNAs can act as competing endogenous RNAs (ceRNAs) to reverse drug resistance and improve therapeutic efficacy, including circRNA-0002483 [233], circRNA-LDB2 [234]and so on. It has been found that anti-PD-1/PD-L1 immuno-resistant cancer cells usually have metabolic changes from oxidative phosphorylation to glycolysis [235]. The excessive lactic acid, as the metabolite of glycolysis in cancer cells, can suppress the function of CD8^+^ T cells, thereby promoting immune escape and anti-PD-1/PD-L1 immuno-resistance [236]. However, metabolic changes occur not only in cancer cells, but also in cancer-associated fibroblasts (CAFs). CAFs, as the essential component of the tumor microenvironment (TME), produce large amounts of lactic acid through aerobic glycolytic metabolism and deliver it to cancer cells, thereby inducing drug resistance [237]. Immunotherapy is a double-edged sword. In clinical trials for lung cancer, anti-PD-1/PD-L1 immunotherapy has been shown to significantly improve survival [238]. Multiple studies have shown a positive correlation between PD-1 mRNA level and the efficacy of anti-PD-1 immune checkpoint inhibitor [239]. Simultaneous inhibition of CTLA-4 and PD-1/PD-L1 pathways results in complementary anti-tumor phenomena, and anti-PD-1 combined with anti-CTLA-4 immunosuppressants clearly prolongs the overall survival of patients with advanced NSCLC [240,241]. However, the upregulation of PD-1 expression may promote the occurrence of tumor immune escape and immune resistance. Some patients develop hyper-progressive disease (HPD) with accelerated tumor growth within a short period of anti-PD-1/PD-L1 treatment [242,243]. Tumor with HPD show reduced immunogenicity, increased immunosuppressive cells, mutations in cancer suppressor genes, and activation of ERK/MAPK, PI3K/AKT, TGF-β, and Insulin-like growth factor 1 (IGF-1) pathways, when compared to tumor without prior HPD [244]. The upstream regulatory mechanism of PD-1/PD-L1 is still largely unknown [120]. With the development and depth of immunotherapy, it is believed that more systematic and comprehensive studies on the regulation mechanisms of PD-1 and PD-L1 will be carried out in the future [71,245].

The abnormal expression of ncRNAs mainly regulates cell proliferation and apoptosis through PI3K/AKT, MAPK and Wnt/β-catenin pathways, which eventually alters the sensitivity of lung cancer cells to chemotherapeutic drugs. PI3K/AKT signaling pathway is aberrantly activated in various cancer types and regulates cancer cells proliferation, metastasis, angiogenesis and metabolism [246,247]. The mTOR, as a common downstream effector molecule of the PI3K/AKT pathway, promotes cells growth and protein synthesis, and phosphorylates the autophagy-related proteins Unc-51 like autophagy activating kinase 1/2 (ULK1/2), resulting in the inhibition of cellular autophagy [195,248,249]. Studies have shown that regulating the activation of the PI3K/AKT/mTOR pathway usually affects the sensitivity of NSCLC cells to DDP [250–252]. Additionally, a variety of anaerobic bacteria are present in the lower respiratory tract of lung cancer patients, and they upregulate the PI3K/AKT pathway to promote the development of lung cancer [253]. There is growing evidence that the microbiota is strongly involved in a number of tumors, including lung cancer [254]. Microbiota and its metabolites modulate cancer-related signaling pathways and ncRNAs to influence autophagy-mediated chemotherapy resistance and tumorigenesis. *Fusobacterium nucleatum* is prevalent in feces and tumor tissues of colorectal cancer patients [255,256], which suppresses the expression of miR-18a* and miR-4802 through activation of the Toll-like receptor 4/Myeloid differentiation primary response protein 88 (TLR4/MYD88) signaling pathway, leading to the up-regulation of autophagy-associated proteins ULK1 and ATG7, eventually activating autophagy and inducing chemotherapy resistance [257]. In gastric cancer, inhibition of autophagy reduces the sensitivity of gastric cancer cells to DDP, miR-21 enhances the activation of PI3K/AKT/mTOR pathway to inhibit autophagy with DDP sensitivity [258]. *Helicobacter pylori* is considered an important carcinogen in the pathogenesis of gastric cancer [259]. When the organism is infected with *Helicobacter pylori*, the autophagy is suppressed through activating the PI3K/AKT/mTOR pathway, which promotes the growth and proliferation of gastric cancer cells [260,261]. Therefore, miR-21 and *Helicobacter pylori* together activate the PI3K/AKT/mTOR pathway, with possible synergistic effects in promoting gastric cancer cells proliferation, inhibiting autophagy and inducing DDP resistance. Studies have shown that ncRNAs (for example, lncR-BC200 [162], lncR-SNHG7 [163]) upregulate the expression of PI3K/AKT to promote DDP resistance in lung cancer. Then, whether ncRNAs can interact with anaerobic bacteria enriched in the lower respiratory tract of lung cancer patients to promote the proliferation, invasion and DDP resistance of lung cancer cells? Not only that, Ge et al. found that HPV-encoded circRNA-E7 could promote immune escape from head and neck squamous cell carcinoma by down-regulating the expression of the immune checkpoint Galectin-9 [262]. EBV is associated with the development of several cancers. In gastric cancer, EBV-encoded miRNA-BART5-5p activates the PIAS3/pSTAT3/PD-L1 axis to significantly enhance PD-L1 expression, leading to an immunosuppressive tumor microenvironment [263]. Similarly, EBV-encoded miRNA-BART11 enhances immune tolerance by up-regulating PD-L1 expression in nasopharyngeal carcinoma cells [264]. Perhaps, in later studies we can focus on the effect of ncRNAs encoded by lung cancer-associated viruses on the immune response.

In summary, ncRNAs, as the regulators of lung cancer drug resistance, play an important role in the process of lung cancer drug resistance by regulating various targets and pathways. Therefore, targeted ncRNA therapies can increase the sensitivity of lung cancer to drugs and further improve patient survival. Many clinical trials of targeted ncRNA therapies are currently underway, but there are still great challenges for clinical application due to safety.

## Data Availability

Data sharing is not applicable to this article as no new data were created or analyzed in this study.
